# Draft Genome Sequence of the Strawberry Anthracnose Pathogen *Colletotrichum fructicola*

**DOI:** 10.1128/MRA.01598-19

**Published:** 2020-03-19

**Authors:** Andrew D. Armitage, Charlotte F. Nellist, Helen J. Bates, Liqing Zhang, Xiaohua Zou, Qing-Hua Gao, Richard J. Harrison

**Affiliations:** a Department of Genetics, Genomics and Breeding, NIAB EMR, Kent, United Kingdom; b Natural Resources Institute, University of Greenwich, Chatham, Kent, United Kingdom; c Shanghai Key Laboratory of Protected Horticultural Technology, Forestry and Fruit Tree Research Institute, Shanghai Academy of Agricultural Sciences, Shanghai, China; d NIAB Cambridge Crop Research, NIAB, Cambridge, United Kingdom; Vanderbilt University

## Abstract

Colletotrichum fructicola is a causal agent of strawberry anthracnose and a major economic pathogen of horticultural and ornamental crops worldwide. Here, we present an annotated draft genome sequence for a *C. fructicola* isolate previously used for transcriptomic analysis. The assembly totals 58.0 Mb in 477 contigs with 18,143 predicted genes.

## ANNOUNCEMENT

Colletotrichum fructicola (phylum *Ascomycota*, class *Sordariomycetes*) is a fungal pathogen of a wide range of horticultural crops, including strawberry, avocado, apple, Asian pear, yam, cacao, coffee, and ornamentals such as statice (1). The fungus is the causative agent of anthracnose on cultivated strawberry in Asia, which leads to severe economic losses (2–4). The hemibiotrophic nature of the fungus has led to recent investigation into gene expression at spore germination, biotrophic and necrotrophic infection (5), and response of the host to infection (6). We provide expansion of the genomic resources available for the anthracnose pathogen *C. fructicola* through assembly and annotation of the same strain used for published transcriptomic work (5).

*C. fructicola* isolate CGMCC3.17371 was isolated in 2007 from an infected strawberry plant displaying strawberry anthracnose symptoms in Feng Xian District, Shanghai. Freeze-dried mycelium, grown in potato dextrose broth (Fluka, Sigma-Aldrich), was used for genomic DNA (gDNA) extraction with a Macherey-Nagel NucleoSpin plant II kit (catalog number 11912262, Fisher). Paired-end PCR-free genomic libraries were prepared for Illumina sequencing using New England Biolabs Next End Repair (catalog number E6050S), dA-tailing (catalog number E6053S), and Blunt T/A ligase (catalog number M0367S) module reagents. For MinION sequencing, library preparation was performed using a 1D genomic DNA ligation sequencing kit (catalog number SQK-LSK108; Oxford Nanopore Technologies), with shearing performed using a g-TUBE (Covaris) and size selection on a BluePippin system (>4 kbp). Genomic libraries were sequenced using Illumina MiSeq v.3 2 × 300-bp paired-end (PE) reads (catalog number MS-102-3003) and MinION FLO-MIN106 R9.4 flow cells, generating 75.49-fold (7,426,411 reads) and 13.57-fold (155,413 reads) coverage of sequence data, respectively.

Illumina reads were trimmed and adapters removed using fastq-mcf v.1.04.676 (7), while MinION reads were trimmed using Porechop v.0.2.0 (https://github.com/rrwick/Porechop). Genome assembly was performed with SPAdes v.3.5.0 (hybridSPAdes) (8), generating a 58-Mb assembly in 447 contigs, with 308 contigs larger than 1,000 bp (Table 1). A total of 2.11 Mb of the genome was repeat masked using RepeatMasker v.4.0.3 (http://www.repeatmasker.org) and TransposonPSI (http://transposonpsi.sourceforge.net; 2013-03-05 release). Genome completeness was assessed using BUSCO v.3 (9), identifying 3,685 of 3,725 (98.9%) genes from the Sodariomycota_*odb9* data set as both present and complete in the assembly. Published RNAseq data from different stages of plant infection (GenBank accession numbers SRR5194993, SRR5194994, and SRR5194995) (5) were aligned to the genome using STAR v.2.5.3a (10). These alignments were used to train prediction of gene models using BRAKER1 v.2.0 using the fungal option and CodingQuarry v.2.0 run in pathogen mode (11, 12). A total of 18,143 genes were predicted, encoding 18,447 proteins, with 16,459 of these proteins predicted from BRAKER1 and supplemented with a further 1,684 genes predicted by CodingQuarry that were located in intergenic regions of BRAKER1 gene models. Functional annotation was performed using InterProScan v.5.18-57.0 (13) and with BLASTp (E < 1 × 10^−100^) searches against the March 2018 release of the SWISS-PROT database (14, 15).

**TABLE 1:** Genome statistics for *Colletotrichum fructicola* isolate CGMCC3.17371

Statistic	Value
Assembly	
Size (bp)	58,056,435
No. of contigs	447
Largest contig (bp)	2,278,540
GC content (%)	53.20
*N*_50_ (bp)	90,947
Repeat masked (%)	3.63
Gene models	
Total no. of genes	18,143
Total no. of proteins	18,447
No. of encoding secreted proteins	2,070
No. of proteins encoding effector candidates	
Secreted carbohydrate active enzymes	516
Secreted with effector-like structure	612

Genes that play a putative role in pathogenicity were identified from predicted gene models. SignalP v.4.1 and TMHMM v.2 were used to predict genes encoding secreted proteins (16, 17), from which carbohydrate-active enzymes were predicted using dbCAN and the CAZy database classification system (18, 19). Furthermore, proteins with an effector-like structure were predicted using EffectorP v.1.0 (20) (Table 1). Default parameters were used for software unless otherwise noted.

### Data availability.

This whole-genome shotgun project has been deposited at DDBJ/ENA/GenBank under the accession number SSNE00000000 (BioProject number PRJNA532407). This version of the project has the accession number SSNE01000000 and consists of sequences deposited under the GenBank accession numbers SSNE01000001 to SSNE01000447. The raw reads are available on the NCBI SRA database under accession number PRJNA532407.

## References

[1] Weir BS, Johnston PR, Damm U 2012 The Colletotrichum gloeosporioides species complex. Stud Mycol 73:115–180. doi:10.3114/sim0011. 23136459 PMC3458417

[2] Xie L, Zhang J, Wan Y, Hu D 2010 Identification of Colletotrichum spp. isolated from strawberry in Zhejiang Province and Shanghai City, China. J Zhejiang Univ Sci B 11:61–70. doi:10.1631/jzus.B0900174. 20043353 PMC2801091

[3] Nam MH, Park MS, Lee HD, Yu SH 2013 Taxonomic re-evaluation of Colletotrichum gloeosporioides isolated from strawberry in Korea. Plant Pathol J 29:317–322. doi:10.5423/PPJ.NT.12.2012.0188. 25288958 PMC4174798

[4] Gan P, Ikeda K, Irieda H, Narusaka M, O’Connell RJ, Narusaka Y, Takano Y, Kubo Y, Shirasu K 2013 Comparative genomic and transcriptomic analyses reveal the hemibiotrophic stage shift of Colletotrichum fungi. New Phytol 197:1236–1249. doi:10.1111/nph.12085. 23252678

[5] Zhang L, Huang X, He C, Zhang Q-Y, Zou X, Duan K, Gao Q 2018 Novel fungal pathogenicity and leaf defense strategies are revealed by simultaneous transcriptome analysis of Colletotrichum fructicola and strawberry infected by this fungus. Front Plant Sci 9:434. doi:10.3389/fpls.2018.00434. 29922301 PMC5996897

[6] Zou X, Guo R, Zhang L, Duan K, Gao Q 2018 Identification of FaNBS-encoding genes responsive to Colletotrichum fructicola infection in strawberry (Fragaria ×ananassa Duchase). Australas Plant Pathol 47:499–510. doi:10.1007/s13313-018-0582-8.

[7] Aronesty E 2013 Comparison of sequencing utility programs. Open Bioinforma J 7:1–8. doi:10.2174/1875036201307010001.

[8] Antipov D, Korobeynikov A, McLean JS, Pevzner PA 2016 hybridSPAdes: an algorithm for hybrid assembly of short and long reads. Bioinformatics 32:1009–1015. doi:10.1093/bioinformatics/btv688. 26589280 PMC4907386

[9] Simão FA, Waterhouse RM, Ioannidis P, Kriventseva EV, Zdobnov EM 2015 BUSCO: assessing genome assembly and annotation completeness with single-copy orthologs. Bioinformatics 31:3210–3212. doi:10.1093/bioinformatics/btv351. 26059717

[10] Dobin A, Davis CA, Schlesinger F, Drenkow J, Zaleski C, Jha S, Batut P, Chaisson M, Gingeras TR 2013 STAR: ultrafast universal RNA-seq aligner. Bioinformatics 29:15–21. doi:10.1093/bioinformatics/bts635. 23104886 PMC3530905

[11] Hoff KJ, Lange S, Lomsadze A, Borodovsky M, Stanke M 2016 BRAKER1: unsupervised RNA-seq-based genome annotation with GeneMark-ET and AUGUSTUS. Bioinformatics 32:767–769. doi:10.1093/bioinformatics/btv661. 26559507 PMC6078167

[12] Testa AC, Hane JK, Ellwood SR, Oliver RP 2015 CodingQuarry: highly accurate hidden Markov model gene prediction in fungal genomes using RNA-seq transcripts. BMC Genomics 16:170. doi:10.1186/s12864-015-1344-4. 25887563 PMC4363200

[13] Jones P, Binns D, Chang H-Y, Fraser M, Li W, McAnulla C, McWilliam H, Maslen J, Mitchell A, Nuka G, Pesseat S, Quinn AF, Sangrador-Vegas A, Scheremetjew M, Yong S-Y, Lopez R, Hunter S 2014 InterProScan 5: genome-scale protein function classification. Bioinformatics 30:1236–1240. doi:10.1093/bioinformatics/btu031. 24451626 PMC3998142

[14] Bairoch A, Apweiler R 2000 The SWISS-PROT protein sequence database and its supplement TrEMBL in 2000. Nucleic Acids Res 28:45–48. doi:10.1093/nar/28.1.45. 10592178 PMC102476

[15] Altschul SF, Gish W, Miller W, Myers EW, Lipman DJ 1990 Basic local alignment search tool. J Mol Biol 215:403–410. doi:10.1016/S0022-2836(05)80360-2. 2231712

[16] Krogh A, Larsson B, von Heijne G, Sonnhammer E 2001 Predicting transmembrane protein topology with a hidden Markov model: application to complete genomes. J Mol Biol 305:567–580. doi:10.1006/jmbi.2000.4315. 11152613

[17] Petersen TN, Brunak S, von Heijne G, Nielsen H 2011 SignalP 4.0: discriminating signal peptides from transmembrane regions. Nat Methods 8:785–786. doi:10.1038/nmeth.1701. 21959131

[18] Huang L, Zhang H, Wu P, Entwistle S, Li X, Yohe T, Yi H, Yang Z, Yin Y 2018 dbCAN-seq: a database of carbohydrate-active enzyme (CAZyme) sequence and annotation. Nucleic Acids Res 46:D516–D521. doi:10.1093/nar/gkx894. 30053267 PMC5753378

[19] Cantarel BL, Coutinho PM, Rancurel C, Bernard T, Lombard V, Henrissat B 2009 The Carbohydrate-Active EnZymes database (CAZy): an expert resource for glycogenomics. Nucleic Acids Res 37:D233–8. doi:10.1093/nar/gkn663. 18838391 PMC2686590

[20] Sperschneider J, Gardiner DM, Dodds PN, Tini F, Covarelli L, Singh KB, Manners JM, Taylor JM 2016 EffectorP: predicting fungal effector proteins from secretomes using machine learning. New Phytol 210:743–761. doi:10.1111/nph.13794. 26680733

